# Stochastic Thermal Properties of Laminates Filled with Long Fibers

**DOI:** 10.3390/ma14102511

**Published:** 2021-05-12

**Authors:** Jan Turant

**Affiliations:** Department of Mechanical Engineering, Informatics and Chemistry of Polymer Materials, Lodz University of Technology, Zeromskiego 116, 90-924 Lodz, Poland; jan.turant@p.lodz.pl

**Keywords:** laminates, effective thermal conductivities, finite element analysis, statistical properties

## Abstract

In this paper, the stochastic parameters of the effective thermal conductivity of multilayer composites are considered. The examined specimens of composites were built with a different number of layers and each had a different saturation density of a composite matrix with fibers. For each case of laminate built with a prescribed number of layers and assumed saturation density, 10,000 tests of its effective thermal conductivity were carried out using numerical experiments. It was assumed that the fibers located in each layer were rectilinear, had a circular cross-section and that they could take random positions in their repeatable volume elements (RVEs). In view of the mentioned assumptions, the heat flux passing throughout a cross-section of a composite sample, perpendicular to the fibers’ direction, was considered. The probability density functions were fitted to the obtained data and then the chosen stochastic parameters of the effective thermal conductivity coefficients were determined.

## 1. Introduction

A material’s properties are determined during physical tests. The kind of test depends on the kind of properties that are being determined. Typical material properties include mechanical, thermal, optical and electromagnetic properties. In the case of composite materials composed of known materials with known properties, virtual tests of their properties can be conducted with accuracy depending on the quality of the physical and mathematical models of a phenomenon and the quality of the solving procedure. It is worth noting that virtual tests of the structural behavior of materials and structures are far cheaper and faster than physical tests carried out in specialized laboratories. Additionally, it should be pointed out that the properties of composite materials cannot be determined with the same precision as typical structurally uniform materials such as steel. The stability of a composite material’s properties is not so obvious and depends not only on the properties of the components but on the arrangement of the components too.

In the presented paper, the stochastic parameters of the effective thermal conductivities of multilayered composites filled with long, parallel, rectilinear fibers with circular cross-sections are considered. The thermal problem is one of the many phenomena described mathematically with such a good approximation that its model can be used in accurate calculations. The process of solving a mathematical model is one of crucial importance and it affects the accuracy of the obtained results. In this case, the finite element method was chosen. The finite element method (FEM) is widely used to solve many engineering problems such as the static and dynamic behavior of mechanical structures, electromagnetics, fluid mechanics and heat flow processes. There are thousands of publications in which authors use and develop this method in all possible fields of its usage.

Particularly in the fields of thermal and coupled thermo-mechanical problems, researchers were able to successfully use the finite element method to show its accuracy in comparison to real experimental data. In article [1], the authors use a finite element model to calculate the strain and residual stress from a temperature field. The results of the numerical experiment were compared with real measurements and showed good compatibility. An analysis of welded parts was carried out in [2]. The authors compared real experimental measurements of deformations with results obtained from the finite element method and obtained a good compliance of results. As another example of successful FEM usage, the problem of multilayered plates built in electron beam additive manufacturing is considered in [3]. In the paper, the authors built a thermo-mechanical model to study the distortion and residual stresses in the plates using this method. The authors compared the results obtained from the finite element analysis with experimental data to ensure the quality of the calculations and to be able to use them in an optimization process.

Numerical tests of the effective thermal conductivities of composite materials are one of the application areas of the FEM. To predict the thermal conductivity of particle-filled epoxy composites, the authors of [4] used the FEM. They built a three-dimensional model that considered the filler geometry and its aspect ratio. Additionally, they used an interfacial layer to simulate the microstructure of epoxy on the fillers. In article [5], the authors developed a new method to generate a three-dimensional FEM model for the calculation of the thermal conductivity with randomly dispersed fillers. In paper [6], a finite element model was developed to calculate the effective thermal conductivity of asphalt concrete with a random aggregate microstructure. The results of the simulation were compared to real experimental data to show that the calculations had a good degree of accuracy. A hybrid finite element method was developed in [7] to evaluate the effective thermal conductivity of fiber-reinforced composites. A numerical evaluation of the effective thermal conductivities of functionally graded fiber materials was used in [8] for the purpose of validating the method proposed for their calculations.

The finite element method is the most accurate method, but a single analysis may be relatively time consuming. In the investigation of effective thermal conductivities, faster methods of estimation can be used (e.g., [9,10]) but we should be aware of their lower accuracy.

The statistical analysis of the properties of composite materials has been analyzed in several articles. In article [11], a stochastic finite element analysis was used to evaluate the probability density of the effective thermal conductivities of composites with randomly distributed spherical particles of different saturations. A stochastic micromechanical model for predicting the probabilistic characteristics of the elastic mechanical properties of a functionally graded isotropic material was considered in [12]. In article [13], investigations into the probabilistic behavior of hybrid fiber-reinforced concrete were carried out. The paper’s authors investigated the behavior of the mechanical properties in the aforementioned material. Studies of the stochastic behavior of the mechanical properties of composites with the inclusion of arbitrary shapes were presented in [14]. Tensile tests were carried out as a result of numerical simulations based on a stochastic finite element analysis.

In the presented paper, the stochastic parameters of effective thermal conductivities are analyzed for the case of a multilayer laminate filled with long, rectilinear fibers with a circular cross-section. An analysis of the effective thermal conductivities was performed on the virtual laboratory stand proposed in [8]. The model of the stand and the tested composite specimen were created using a finite element model. It was assumed that each layer of the laminate is composed of repeatable volume elements in which a fiber can take any position with uniform probability. The calculations were conducted for laminates made with different numbers of layers and different composite saturations of fibers. For each tested type of laminate (with a different saturation of fibers and a different number of layers), 10,000 numerical experiments were carried out. Next, the probability distribution for effective thermal conductivity was fitted, and the chosen stochastic parameters were calculated. All of the necessary stages of executed procedures were applied using the FORTRAN programming language.

## 2. Problem Formulation

**Figure 1:**
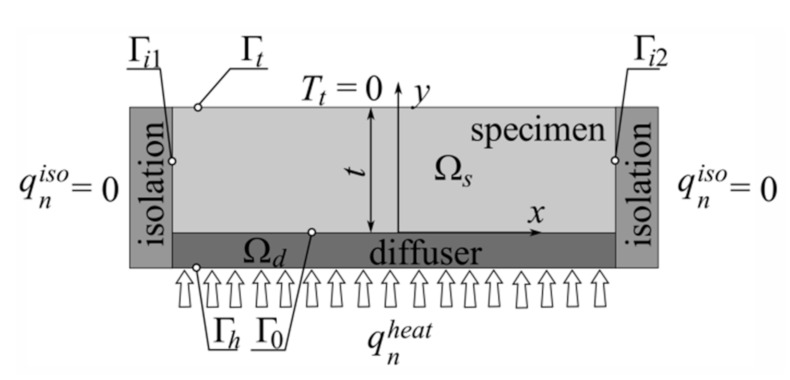
Schema of the virtual stand.

Figure 1 shows the schema of the virtual stand that was used to evaluate the effective thermal conductivities of laminate. Let us consider two-dimensional steady-state heat transfer for a laminate of thickness *t* built with several layers (specimen) and a diffuser whose only task is to equalize the temperature on the lower surface Γ_0_ of the specimen in order to simplify further calculations. To describe the behavior of the structure at each point, we can use some typical relations described by the following heat equation and Fourier’s law [15]:

(1) $${divq+f=0,\;q=-\lambda \nabla T\;\mathrm{in}\;\;\;\;\;\;\Omega }_{s},\;\Omega_{d}$$

where **q** and *f* denote a heat flux intensity and a heat source, respectively; **λ** is the matrix of thermal conductivity coefficients and ∇*T* denotes the gradient of the temperature field.

To be able to solve this type of problem, we have to determine the proper boundary conditions characteristic of the given environmental conditions, such as temperature, heat flux intensity or convection on the proper parts of a boundary (Figure 1). The mentioned conditions, in the case shown in Figure 1, can be written in the following form:

(2) $$T=T_{t}\;\mathrm{on}\;\Gamma_{t},\;\;\;\;\;q_{n}=q\cdot n=q_{n}^{iso}\;\mathrm{on}\;\Gamma_{i1}\cup \Gamma_{i2},\;\;\;\;\;q_{n}=q\cdot n=q_{n}^{heat}\;\mathrm{on}\;\Gamma_{h}$$

where *T_t_*, *q_n_^iso^* and *q_n_^heat^* denote the prescribed values of temperature and the heat fluxes, and **n** is the normal unit vector of the boundary line at a chosen point. By considering the heat flux in the *y* direction, following 1^2^ and taking into account the uniform temperature along the boundary Γ_0_, we can write the following:

(3) $$\lambda_{ey}=\frac{q_{n}^{heat}t}{T_{0}}$$

where *λ_ey_* denotes the wanted effective thermal conductivity in the *y* direction and *T*_0_ is the temperature (along boundary Γ_0_) that should be determined during the FEM calculations.

## 3. Methods

### 3.1. Finite Element Model

**Figure 2:**
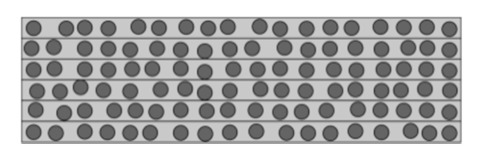
Cross-section of a specimen.

The specimen’s inner structure consisted of several layers of composite filled with long, rectilinear fibers with a circular cross-section. The structure of the specimen’s cross-section perpendicular to the fiber’s axis is shown in Figure 2.

**Figure 3:**
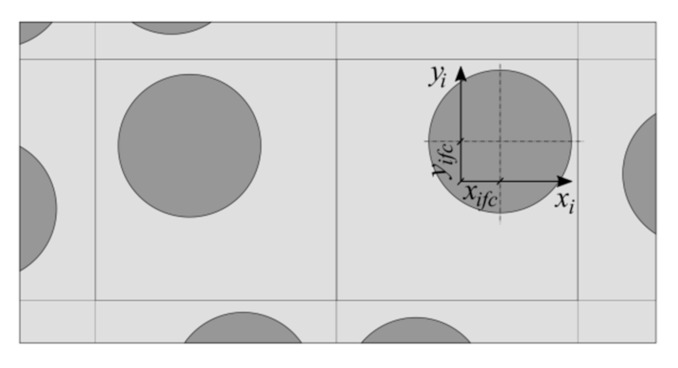
Repeatable volume element.

It was assumed that each layer of the laminate is built of many repeatable volume elements in which the fiber can take any position (Figure 3).

Furthermore, it was assumed that the RVE is square in shape and that the position of the fiber cross-section center (*x_ifc_*, *y_ifc_*) was randomly chosen.

The behavior analysis of the specimen and of the whole virtual stand was made using the FEM. All of the necessary procedures were written in FORTRAN programming language. The basics of the FEM have been discussed in many publications. The most important and well known are publications written by Zienkiewicz et al. (e.g., [16]). In the aforementioned publication, the basis for solving heat conduction problems as well as general investigations into automatic mesh generation can be found. Additionally, the book provides information about errors within the method and its convergence criteria. A detailed engineering approach to heat transfer can be found in [17].

**Figure 4:**
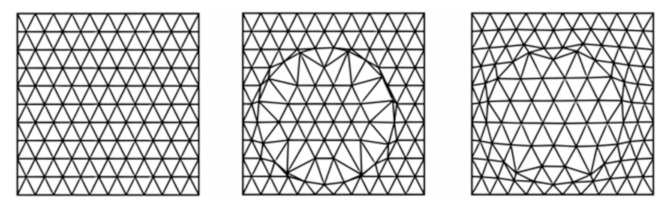
Three-step process of mesh generation.

The discretization of the structure domain is one of the crucially important stages of the FEM. In the presented paper, a dedicated algorithm for automatic mesh generation was created to accelerate the process of its creation. First, an area of the specimen was discretized with a regular mesh of triangles. Next, the nodes of the mesh closest to the fiber’s cross-section boundary were moved to the boundary line. Finally, Laplacian smoothing of the mesh was carried out. The three-step process of mesh generation for one RVE is shown in Figure 4.

**Figure 5:**
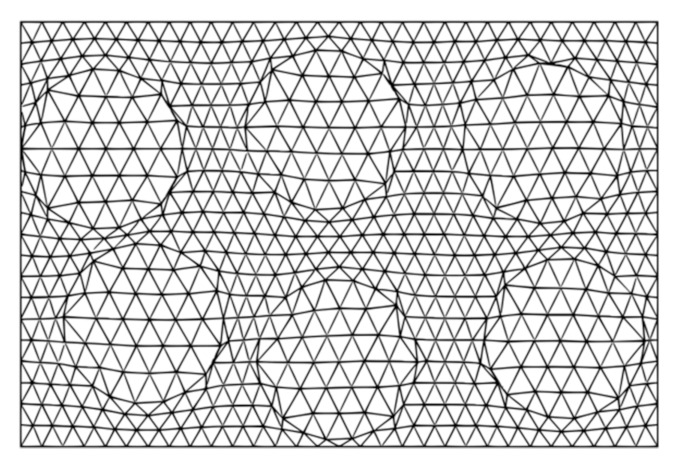
Mesh of the specimen built with 2 layers and 3 RVEs in each layer.

The mesh of a specimen built with 2 layers consisting of 3 RVEs each and obtained using this technique is shown in Figure 5.

The calculations were carried out for a laminate built with 2 to 8 layers, while the number of RVEs was always equal to 10 in each layer. For each number of layers, the various saturation densities of the matrix with fibers were examined. The tests for saturation density were executed from the interval [0.25, 0.55] in steps of 0.05. In each case, the dimension of the square RVE was assumed to be 0.001 (*m*). The dimensions of the filled fiber cross-section result from the required saturation density with fibers. The fiber and the matrix were assumed to be thermally isotropic. The conductivity coefficients were assumed to be 1, 20 and 2 × 10^6^ W/(mK) for the matrix, the fiber and the diffuser materials, respectively. The diffuser material was artificial. The acting heat flux *q_n_^heat^* was equal to 100 W/m^2^. For each of the examined numbers of layers and saturation densities, 10,000 virtual specimens were created and tested. Each separate RVE was discretized with 820 triangular elements, which means that in the case of a specimen built with 8 layers, 65,600 triangular finite elements were used to analyze the specimen’s behavior. The obtained results were the basis for a further investigation, during which the best fitted probability distributions were chosen.

### 3.2. Probability Distribution Fitting

With respect to the observed right-skewed distribution of the measured data, some probability distributions of this type were chosen to be fitted to the obtained data. The three following distributions were chosen: log-logistic, log-normal and gamma [18].

#### 3.2.1. Log-Logistic Distribution

The probability density function is as follows:

(4) $$f(x)=\frac{(\beta /\alpha ){\left(x/\alpha \right)}^{\beta -1}}{{\left(1+{\left(x/\alpha \right)}^{\beta }\right)}^{2}}$$

where *β* is the shape parameter, *α* is the scale parameter and *x* ∈ [0, ∞).

#### 3.2.2. Log-Normal Distribution

The probability density function is as follows:

(5) $$f(x)=\frac{1}{x\beta \sqrt{2\pi }}e^{-\frac{{\left(\ln x-\alpha \right)}^{2}}{2\beta^{2}}}$$

where *α* is the location parameter, *β* is the shape parameter and *x* ∈ [0, ∞).

#### 3.2.3. Gamma Distribution

The probability density function is as follows:

(6) $$f(x)=\frac{1}{\Gamma (\beta )\alpha^{k}}x^{\left(\beta -1\right)}e^{-x/\alpha }$$

where *β* is the shape parameter, *α* is the scale parameter, Γ(*β*) denotes the gamma function and *x* ∈ [0, ∞).

#### 3.2.4. Fitting Process

The number of classes of a histogram was established according to Scott’s rule [19]. To fit a proper probability function to the experimental data, the least squares method was chosen. In the minimization procedure, necessary during this stage, the evolutionary floating-point algorithm was used. In evolutionary algorithms, the improvement of the solution is obtained during a process similar to a natural evolution. The typical stages of evolution are selection, crossover and mutation of individuals (solutions). In the case of the used algorithm, tournament selection, heuristic crossover and non-uniform Gaussian mutation were applied [20]. The parameters of distribution (*α, β*) and its shift (*σ*) were assumed as the design variables. Consequently, the vector of design variables was **v** = {*α*,*β*,*σ*}^T^ and it was treated as an individual of a population during the evolutionary process. According to the used fitting method, the following function was chosen as a fitness function:

(7) $$ff(v)=\sqrt{\sum_{i=1}^{m}{\left(y_{id}-y_{ie}\right)}^{2}}$$

where *m* denotes the number of classes of the histogram, and *y_id_* and *y_ie_* are values of the probability density function and the frequency of occurrence of an effective thermal conductivity in the i-th class, respectively.

## 4. Results

**Figure 6:**
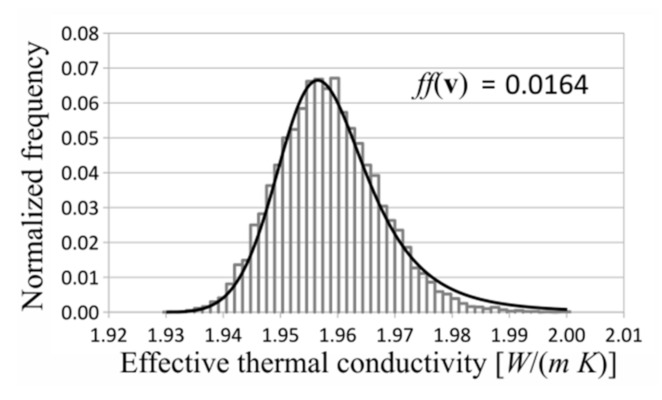
Histogram and log-logistic distribution.

**Figure 7:**
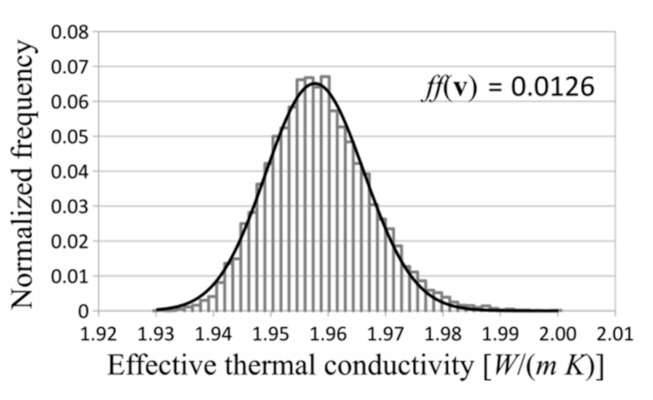
Histogram and log-normal distribution.

**Figure 8:**
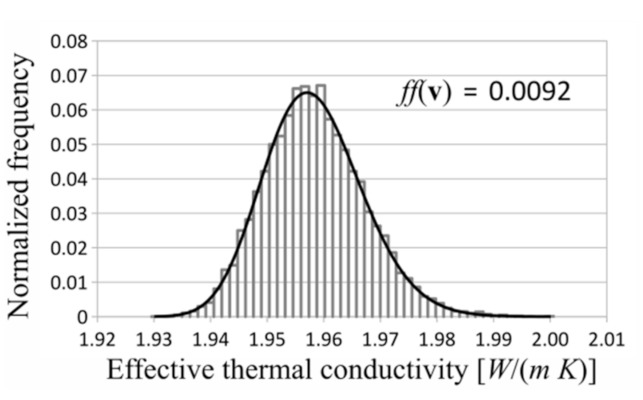
Histogram and gamma distribution.

In each investigated case, the gamma distribution turned out to be the best fitted distribution to the measurement data. Exemplary investigated distributions and histograms are shown in Figure 6, Figure 7 and Figure 8. The shown distributions were created with the following parameters: saturation density, 0.35; number of layers, 5; and number of RVEs, 10 in each layer.

To evaluate the most probable magnitude of an effective thermal conductivity, the modes were calculated in each investigated case of the laminate. Taking into account the shift *σ*, the mode for gamma distribution is expressed as follows:

(8) mode=(β−1)α+σ

To estimate the stability of the measured quantity, the standard deviation was calculated. The standard deviation for gamma distribution is expressed as follows:

(9) $$standard\_deviation=\alpha \sqrt{\beta }$$

All of the calculated modes and standard deviations for the effective thermal conductivities are given in Table 1, Table 2, Table 3, Table 4, Table 5, Table 6 and Table 7.

**Figure 9:**
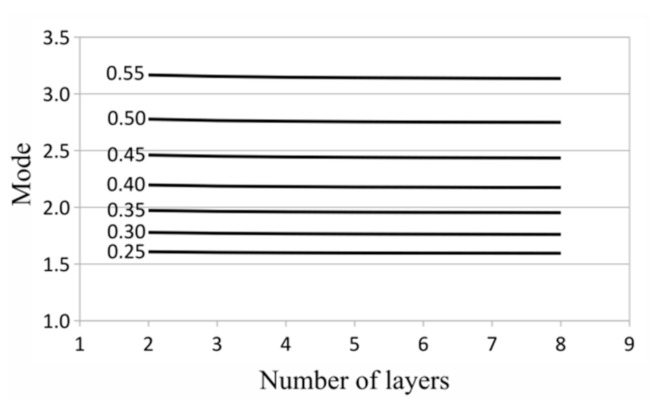
Modes for different numbers of layers and a different saturation density of matrix with fibers.

**Figure 10:**
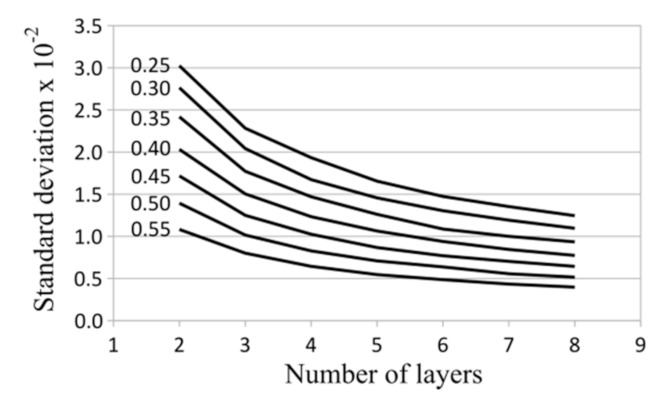
Standard deviations for different numbers of layers and a different saturation density of matrix with fibers.

The plot of modes is presented in Figure 9 and the plot of standard deviations is presented in Figure 10.

## 5. Discussion

This paper is the first publication to deal with the stability of effective thermal conductivities in the case of laminates filled with long, parallel fibers with a circular cross-section.

In each case of saturation density, the modes of gamma distribution slightly decreased with the increasing number of laminate layers (Figure 9). The stability of the values (determined by the standard deviation) of measured effective thermal conductivities always increased with the number of laminate layers and the saturation density of the matrix with fibers (Figure 10).

The higher stability of magnitudes of the effective thermal conductivities for a larger number of layers is understandable and can be explained by the lower probability of achieving a particular, less uniform arrangement of fibers. Similarly, the greater saturation density of the matrix with fibers caused a higher predictability of the effective thermal conductivities, which stemmed from the more limited number of fiber positions.

It is worth emphasizing that the mean values depend on the number of layers. The effective thermal conductivities for a greater number of layers were slightly lower for each case of the saturation density of the matrix with fibers (in these calculations, the thermal conductivity coefficient of the matrix was lower than the coefficient of the fiber). Particularly, the mean of the effective thermal conductivities for an eight-layer laminate was about 1% lower than that for a two-layer laminate and 0.2% lower than for a four-layer laminate in all of the investigated cases of matrix saturation. 

The modes of the observed quantity were of the zero order, while correspondent standard deviations were, on average, of the order −2. It should be noted that all of the expected values of thermal conductivities differ from the mean by no more than 1% on average. The highest observed average difference, following from the standard deviation, was about 1% of the mean (for two layers and a high saturation density) and the lowest was about 0.3% (for eight layers and a low saturation density).

## 6. Conclusions

The simulation methods for investigating the behavior of materials give a great opportunity for the statistical analysis of a composite material’s properties. The properly prepared and executed numerical experiment gives results similar to those from a real experiment but is much cheaper and faster. The statistical analysis requires the preparation of no less than 30 specimens, but the more specimens that are prepared and examined, the higher the accuracy of the statistical analysis will be. Generally, a higher number of tests means a higher quality of statistical investigation. In the case of virtual experiments, it is possible to automate the whole process of virtual specimen creation, its behavior analysis and its statistical analysis. It is worth noting that the behavior analysis can be paralleled. Many single analyses can be carried out on many computers and their processors. In the case of the presented research, the analyses were carried out using 15 personal laboratory computers with central processor units consisting of four processors, which meant that 60 numerical tests could be carried out at the same time. To complete all the tests, almost half a million specimens were examined, which is an unreachable value for tests made on real specimens.

For the investigated laminates and probability distribution functions, the gamma distribution turned out to be best fitted to obtain results of the behavior of the effective thermal conductivities. 

The obtained results show the character of the stochastic behavior of the thermal conductivities of laminates of a given class and can be used to confirm their relative stability, regardless of the number of layers.

To perform many of the tasks necessary to analyze the stochastic behavior of the thermal conductivities of laminates, the fast dedicated mesh generation method was proposed and an attempt to find the proper probability distribution was carried out without an arbitrary assumption of its normal character, which is typical for this type of rare investigation. The described procedures for obtaining the stochastic behavior of the thermal conductivities of laminates can be used for many cases of composite materials.

## Figures and Tables

**Table 1:** Modes and standard deviations for the saturation density of 0.25 and the given number of layers.

Number of Layers	Modes	Standard Deviations
2	1.609	1.08 × 10^−2^
3	1.602	8.00 × 10^−3^
4	1.599	6.44 × 10^−3^
5	1.597	5.46 × 10^−3^
6	1.596	4.86 × 10^−3^
7	1.596	4.34 × 10^−3^
8	1.595	3.97 × 10^−3^

**Table 2:** Modes and standard deviations for the saturation density of 0.30 and the given number of layers.

Number of Layers	Modes	Standard Deviations
2	1.778	1.39 × 10^−2^
3	1.771	1.01 × 10^−2^
4	1.767	8.27 × 10^−3^
5	1.765	7.09 × 10^−3^
6	1.764	6.37 × 10^−3^
7	1.763	5.56 × 10^−3^
8	1.762	5.16 × 10^−3^

**Table 3:** Modes and standard deviations for the saturation density of 0.35 and the given number of layers.

Number of Layers	Modes	Standard Deviations
2	1.972	1.72 × 10^−2^
3	1.964	1.25 × 10^−2^
4	1.960	1.02 × 10^−2^
5	1.957	8.67 × 10^−3^
6	1.955	7.69 × 10^−3^
7	1.954	7.03 × 10^−3^
8	1.953	6.43 × 10^−3^

**Table 4:** Modes and standard deviations for the saturation density of 0.40 and the given number of layers.

Number of Layers	Modes	Standard Deviations
2	2.197	2.03 × 10^−2^
3	2.187	1.50 × 10^−2^
4	2.183	1.23 × 10^−2^
5	2.179	1.06 × 10^−2^
6	2.178	9.39 × 10^−3^
7	2.176	8.44 × 10^−3^
8	2.175	7.73 × 10^−3^

**Table 5:** Modes and standard deviations for the saturation density of 0.45 and the given number of layers.

Number of Layers	Modes	Standard Deviations
2	2.461	2.42 × 10^−2^
3	2.450	1.77 × 10^−2^
4	2.444	1.47 × 10^−2^
5	2.441	1.26 × 10^−2^
6	2.438	1.09 × 10^−2^
7	2.437	1.00 × 10^−2^
8	2.436	9.34 × 10^−3^

**Table 6:** Modes and standard deviations for the saturation density of 0.50 and the given number of layers.

Number of Layers	Modes	Standard Deviations
2	2.778	2.76 × 10^−2^
3	2.765	2.04 × 10^−2^
4	2.759	1.67 × 10^−2^
5	2.755	1.46 × 10^−2^
6	2.752	1.30 × 10^−2^
7	2.751	1.19 × 10^−2^
8	2.749	1.10 × 10^−2^

**Table 7:** Modes and standard deviations for the saturation density of 0.55 and the given number of layers.

Number of Layers	Modes	Standard Deviations
2	3.167	3.02 × 10^−2^
3	3.154	2.28 × 10^−2^
4	3.147	1.93 × 10^−2^
5	3.143	1.66 × 10^−2^
6	3.140	1.47 × 10^−2^
7	3.138	1.35 × 10^−2^
8	3.136	1.24 × 10^−2^

## Data Availability

The raw data required to reproduce these findings are available on request from the corresponding author.
